# Identification of Candidate Target Genes and Immune Cells in Oral Squamous Cell Carcinoma

**DOI:** 10.1155/2021/5802110

**Published:** 2021-12-30

**Authors:** Pengfeng Xie, Shichao Wu, Lijuan Guo, Jun Ren, Kaizhi Cai, Mingyue Zhou, Weiwei Liu, Sen Yang

**Affiliations:** ^1^Special Treatment, Jinan Stomatological Hospital, Jinan, Shandong, China; ^2^Department of Prosthodontic, Tianjin Binhai New Area Tanggu Stomatology Hospital, China; ^3^Department of Oral and Maxillofacial Surgery, Suining Central Hospital, China; ^4^School of Stomatology, Jining Medical College, Shandong Province, China

## Abstract

**Background:**

The advance of new treatment strategies for more effective management of oral cancer requires identification of novel biological targets. Therefore, the purpose of this study is to identify novel biomarkers associated with oral tumorigenesis and prognostic signature by comparing gene expression profile of oral squamous cell carcinomas (OSCCs).

**Methods:**

Four datasets including GSE25099, GSE30784, GSE37991, and GSE41613 were collected from Gene Expression Omnibus (GEO) database. Gene Ontology (GO) and the Kyoto Encyclopedia of Genes and Genomes (KEGG) analysis, Cox model analysis, identification of key genes, and Kaplan-Meier analysis were also performed. The xCell was utilized to analyze the infiltration levels of immune cells.

**Results:**

A total of 235 differentially expressed genes (DEGs) were found to be dysregulated in OSCC. These genes were mainly enriched in ECM receptor interaction and focal adhesion. Cox regression analysis identified 10 genes considered as key genes. Kaplan-Meier analysis showed that low expression of SERPINE1 (also known as PAI-1), high expression of CD1C, and C-X3-C motif chemokine receptor 1 (CX3CR1) were associated with well prognostic status in OSCC patients. In addition, we constructed a 3-immune-cell signature (myeloid dendritic cell, T cell CD4^+^ central memory, and common myeloid progenitor) that may be used to predict the survival status of OSCC patients.

**Conclusion:**

Three key genes and 3-immune-cell signature were potential biomarkers for the prognosis of OSCC, and they may serve as potential targets for the treatment of OSCC patients.

## 1. Introduction

Head and neck cancers are mostly derived from oral, pharyngeal, and laryngeal mucosal epithelia, collectively referred to as head and neck squamous cell carcinoma [1]. Oral squamous cell carcinoma (OSCC) is one of the more common 10 malignant tumors in humans, accounting for 40% of head and neck squamous cell cancer (HNSCC) [2]. Approximately 350 000 people are diagnosed with oral squamous cell carcinoma each year [3]. Current treatment of OSCC mainly relied on single or multimodality therapy including surgery, radiation, and/or chemotherapy. Despite rapid advances in these treatment modalities as well as improvements in the early detection of oral cancer, the 5-year survival rate of OSCC remains only 50% in the past decades and 25%–50% of them will suffer from local relapse and distant metastasis after treatment [4]. The incidence of OSCC is high and the prognosis is poor, and an increasing number of investigators have conducted in-depth studies on the pathogenesis and potential therapeutic targets for OSCC [5].

Despite there have been several articles reporting the discovery of OSCC biomarkers, only few of them have been validated and successfully applied in routine clinical practice [1–3, 6, 7]. Nonetheless, there were some limitations with regard to most biomarkers in the early detection of OSCC, and sometimes, the prognostic value of them was inaccurate that plagued most people [7]. By using bioinformatics analysis, it is more likely to overcome the defects in the reported biomarkers. Bioinformatics analysis enabled researchers to in-depth study the comprehensive data of a large number of clinical samples from different independent studies, which provided a data infrastructure for unraveling promising biomarkers and updating our understanding of cancer [3, 5, 6].

The tumor microenvironment (TME) is a complex and heterogeneous mix of tumor cells and stromal cells, including endothelial cells, cancer-associated fibroblasts (CAFs), and immune cells [8]. The TME assists in tumor initiation and progression [9]. CAFs further assist oral squamous cell carcinoma metastasis to lymph nodes because of the infiltration of immune heterogeneous immune cells [10]. OSCC has lymph node metastasis in more than 40% of patients at the time of diagnosis, and worse, lymph node metastasis has decreased the survival rate of OSCC patients by nearly 50% [11].

All malignancies have the special ability to allow continued proliferation, invasion, and metastasis. Pathophysiological processes are driven by some complex molecular signaling pathways [12]. There have been numerous studies showing that aberrant expression of genes in OSCC affects prognosis and is implicated in the alteration of multiple biological functions [13]. However, specific molecular OSCC prophecies have only been partially identified [14]. It is therefore necessary to search for effective diagnostic and prognostic biomarkers for OSCC.

Here, we utilized sequencing data of OSCC from public databases and analyzed potential genes for prognosis and identified abnormally regulated biological functions. Additionally, we employed the evaluation of immune cell infiltration to judge the prognosis of OSCC.

## 2. Materials and Methods

### 2.1. Data Collection

GSE25099 included gene expression profile of oral tissue from 57 specimens from patients with OSCC and 22 oral tissues from normal persons. GSE30784 included gene expression profile of oral tissues from 167 OSCC and 45 normal controls. GSE37991 included gene expression profile of biopsies from 40 OSCC patients and 40 nontumor pairwise samples. Survival data for OSCC patients were obtained from GSE41613. These data were all collected from the Gene Expression Omnibus (GEO) database.

### 2.2. Differential Analysis

Differential expression analysis was performed using the limma R package. The genes with an ∣log2(FoldChange) | >1 and *P* < 0.05 between OSCC and control were assigned as differentially expressed genes (DEGs).

### 2.3. Enrichment Analysis

The enrichment analysis of Gene Ontology (GO) and Kyoto Encyclopedia of Genes and Genomes (KEGG) for common genes was analyzed using the Enrichr R package. The *P* value < 0.05 was considered significantly enriched.

### 2.4. Immune Cell Infiltration

The xCell R package [15] was used to assign and visualize 39 types of immune cells in OSCC and controls.

### 2.5. Identification of Risk Prognostic Factors

Genes with significant prognostic value (*P* < 0.05) in DEGs or immune cells were determined with univariate Cox regression analysis. OSCC samples were divided into high-risk and low-risk groups based on the median level of Cox score. The accuracy of the Cox score was assessed through receiver operating characteristic (ROC) curve with the pROC R package. Then, the prognostic value of high-risk or low-risk groups was assessed using the Kaplan-Meier (KM) curves through the survival R package.

## 3. Results

### 3.1. Genes Associated with OSCC

**Figure 1:**
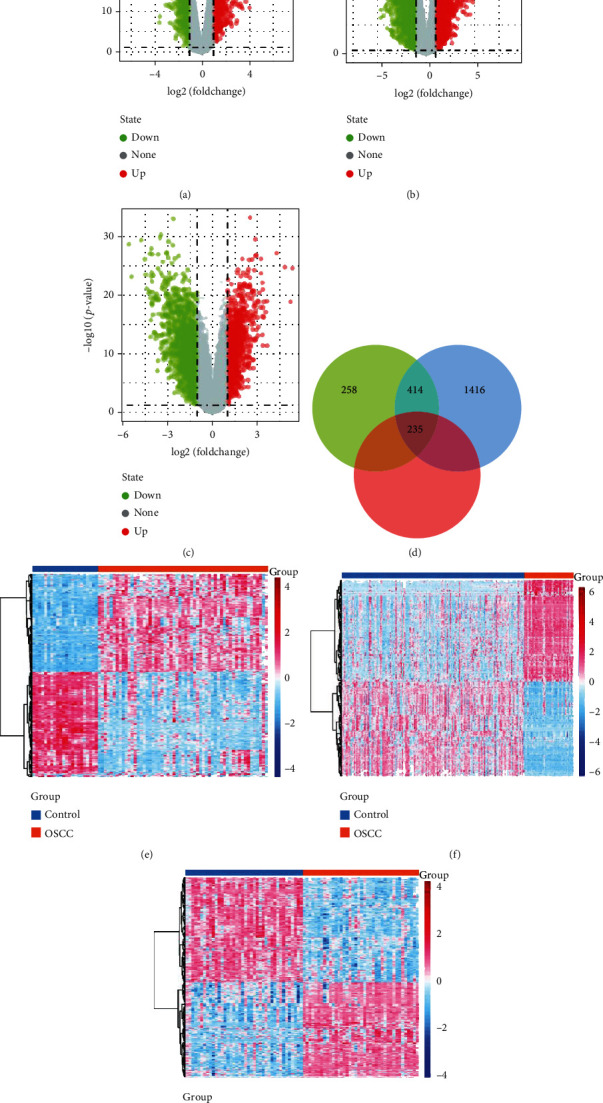
Identification of deregulated expressed genes for oral squamous cell carcinoma. Differentially expressed genes between OSCC and controls in (a) GSE25099, (b) GSE30784, and (c) GSE37991. (d) Venny map of differentially expressed genes among the three groups of DEGs. Heatmap of common genes in (e) GSE25099, (f) GSE30784, and (g) GSE37991. OSCC: oral squamous cell carcinoma.

To identify OSCC-associated genes, the differential analysis of gene expression between OSCC and controls was performed. We obtained 973 DEGs in GSE25099 (Figure 1(a)), 2488 DEGs in GSE30784 (Figure 1(b)), and 2204 DEGs in GSE37991 (Figure 1(c)). We identified 235 common genes among the three sets of DEGs by intersection analysis (Figure 1(d)). The common genes were similarly aberrantly expressed in GSE25099 (Figure 1(e)), GSE30784 (Figure 1(f)), and GSE37991 (Figure 1(g)).

### 3.2. Molecular Mechanisms of OSCC

**Figure 2:**
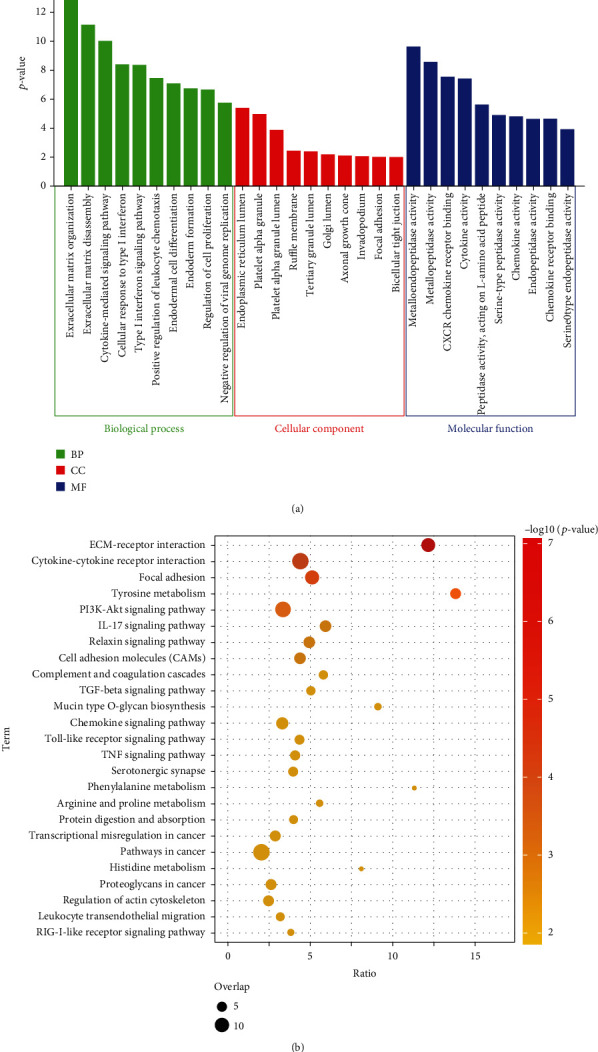
Enrichment analysis of common genes. (a) The top significant 10 terms of biological processes, cellular components, and molecular functions of common gene enrichment. (b) KEGG pathways significantly enriched in common genes.

To identify the molecular mechanisms associated with OSCC, we performed an enrichment analysis of common genes. GO results (Figure 2(a)) showed that these genes were mainly enriched in extracellular matrix organization, cytokine-mediated signaling pathway, and response to type I interferon of biological processes (BP). Endoplasmic reticulum lumen and platelet alpha granule were significantly enriched in cellular components (CC). And metalloendopeptidase activity and metallopeptidase activity of molecular functions (MF) were enriched by common genes. In addition, ECM receptor interaction, cytokine receptor interaction, and focal adhesion were significantly enriched by common genes in KEGG enrichment results (Figure 2(b)).

### 3.3. Candidate Genes for OSCC

**Figure 3:**
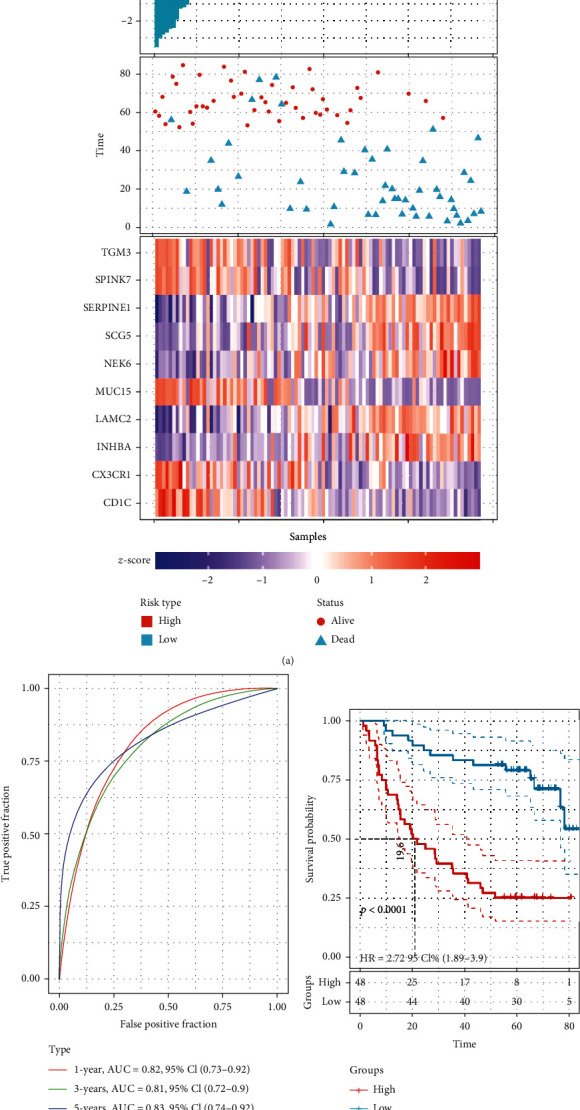
Risk score analysis of gene signature in OSCC. (a) Risk score and survival status distribution according to the median of the risk score for each signature. (b) ROC curves of risk scores to predict patients' 1-, 3-, and 5-year survival. (c) Kaplan-Meier survival curves of high- and low-risk groups for OSCC patients. OSCC: oral squamous cell carcinoma.

**Figure 4:**
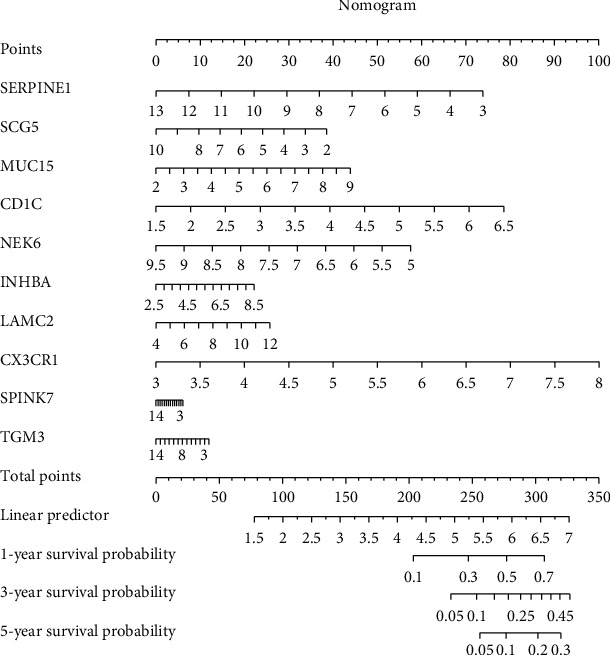
Nomogram of risk gene signature.

To identify candidate target genes for OSCC, we performed Cox regression analysis on common genes. The top 10 most significant genes were selected as key genes (Table 1). The OSCC samples were divided into high-risk and low-risk groups by the median value of the risk score (Figure 3(a)). In the high-risk group, there were fewer OSCC patients ultimately surviving, compared with the low-risk group. The expression of key genes differed in the high-risk and low-risk groups. In addition, the risk score had a good ability to predict the prognosis of OSCC patients (AUC > 0.8) (Figure 3(b)). The low-risk group had a significantly higher survival probability than the high-risk group (Figure 3(c)). The nomogram results showed that low expression of SERPINE1, high expression of CD1C, and CX3CR1 may benefit the survival probability of OSCC patients (Figure 4).

### 3.4. Immune Infiltration of OSCC

**Figure 5:**
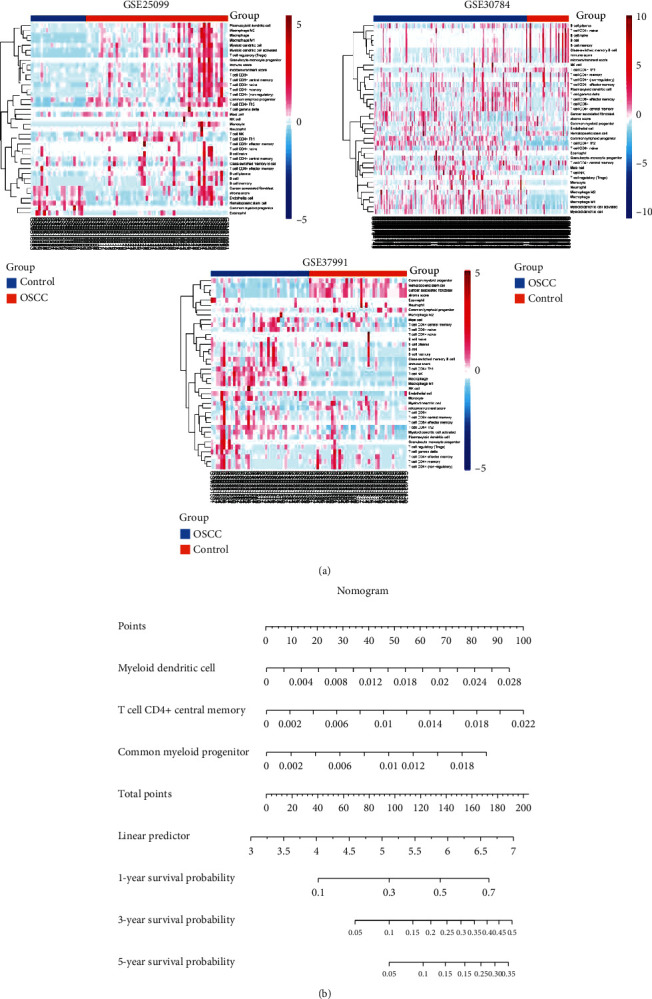
Immune cell infiltration in OSCC. (a) Heatmap of immune cell infiltration in OSCC and control of GSE25099, GSE30784, and GSE37991. (b) Nomogram of risk immune cell signature. OSCC: oral squamous cell carcinoma.

**Figure 6:**
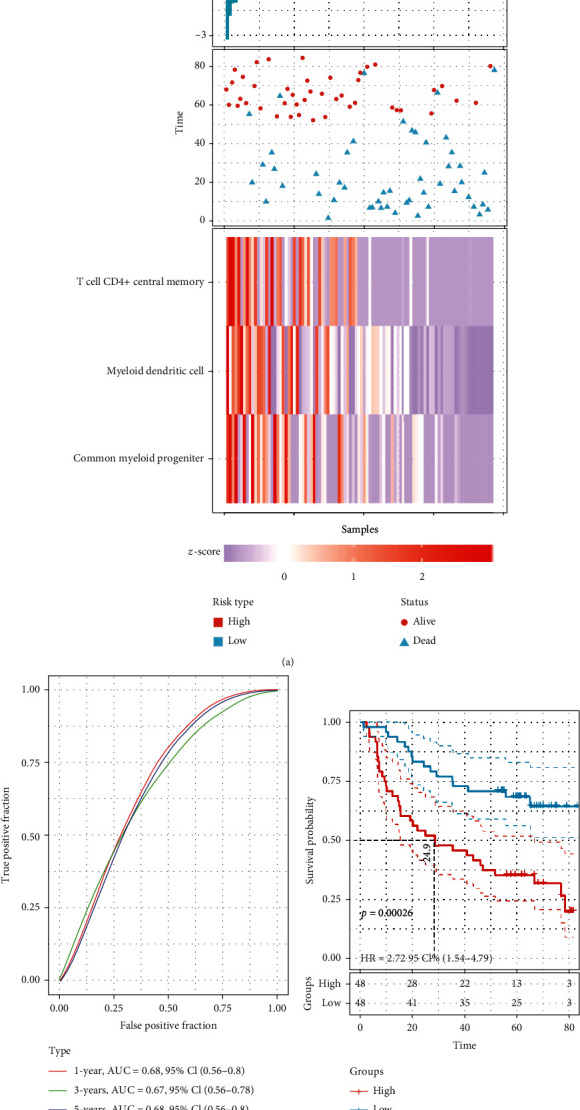
Risk score analysis of immune cell signature in OSCC. (a) Risk score and survival status distribution according to the median of the risk score for immune cell signatures. (b) ROC curves of risk scores to predict patients' 1-, 3-, and 5-year survival. (c) Kaplan-Meier survival curves of high- and low-risk groups for OSCC patients. OSCC: oral squamous cell carcinoma.

Among the signaling pathways enriched in common genes, we found a large number of immune inflammation-related pathways. Therefore, we calculated the infiltration of immune cells in OSCC patients (Figure 5(a)). Cox regression analysis identified three immune cells that may be significant for the prognosis of OSCC patients (*P* < 0.05). Nomogram results showed that high infiltration levels of myeloid dendritic cell, T cell CD4^+^ central memory, and common myeloid progenitor were beneficial to patient prognosis (Figure 5(b)). OSCC patients were divided into high-risk and low-risk groups by the median values of the risk scores for the three immune cells (Figure 6(a)). In the high-risk group, there were fewer OSCC patients ultimately surviving, compared with the low-risk group. The infiltration of immune cells differed in the high-risk and low-risk groups. The risk score had a potential ability to predict the prognosis of OSCC patients (AUC > 0.6) (Figure 6(b)). In addition, the low-risk group had a significantly higher survival probability than the high-risk group (Figure 6(c)).

## 4. Discussion

Our study utilized different public data to analyze the genes differentially expressed in OSCC and investigate the related signaling pathways to identify possible biological roles and target genes in the process of OSCC. Immune cell infiltration levels in OSCC were also analyzed utilizing public data. Cox regression analysis was further utilized to identify candidate genes and immune cells that may influence the prognosis of OSCC. In addition, we also found abnormal immunoinflammatory signaling pathways in OSCC.

Utilizing three datasets, we identified common genes that were differentially expressed between OSCC and controls. The enrichment results of these genes revealed that OSCC-related genes were mainly involved in extracellular matrix organization, response to type I interferon, and cell proliferation. We identified important pathways in KEGG results. This is consistent with previous studies. The extracellular matrix has been reported to be potentially involved in the progression and spread of OSCC [16, 17]. Type I interferon, as an autocrine or paracrine pathway underlying cancer immunosurveillance, is involved in the immune response process of OSCC [18, 19]. ECM remodeling can promote tumor growth and invasiveness, with a key role in oral squamous cell carcinogenesis and progression [20, 21]. The formation of ECM receptor interaction and focal adhesion signaling pathways may be associated with integrin gene expression and contribute to OSCC progression [22, 23]. Focal adhesion is a cell substratum adhesion structure mediated by integrins, whose functions include immobilizing the ends of actin filaments, facilitating strong attachment to substrates, and functioning as an integrin signaling platform [24].

The results of Cox regression analysis and nomogram showed that the abnormal expression of SERPINE1, CD1C, and CX3CR1 might affect the prognosis of OSCC patients. SERPINE1 is an important gene associated with metastasis and promotes OSCC invasion and metastasis [25]. SERPINE1, also known as plasminogen activator inhibitor-1, is a regulator of the urokinase and tissue-type plasminogen activators. These serine proteases in turn activate the proenzyme plasminogen to plasmin that promotes invasion by degradation of the extracellular matrix, as well as activation of matrix metalloproteinases. SERPINE1 is a potential prognostic tool in OSCC through cell adhesion, migration, invasion, and cell proliferation [26]. CD1C is a relevant gene for dendritic cells and is involved in the immune microenvironment of OSCC [27]. Dendritic cell subtype which highly expresses CD1C can elicit a wide range of responses, including activation of Th1 and Th2 [28]. The gene coding for C-X3-C motif chemokine receptor 1 (CX3CR1) is located on chromosome 3p22.2. CX3CR1 is a 7-transmembrane receptor coupled to heterotrimeric G proteins (GPCRs). The CX3CL1-CX3CR1 axis mediates the adhesion of leukocytes and is also involved in cell survival and recruitment of immune cell subpopulations. Downregulation of CX3CR1 can significantly inhibit lung cancer and pancreatic ductal adenocarcinoma cell proliferation and increase apoptosis [29, 30]. Therefore, we conclude that SERPINE1, CD1C, and CX3CR1 are therapeutic targets for OSCC and have future clinical implications. As previously described, a set of prognostic signatures including PLAU, CLDN8, and CDKN2A was identified from the database to predict overall survival in patients with OSCC. PSMA7, ITGA6, ITGB4, and APP were overexpressed in HNSCC, and they exhibited a significant correlation with poor overall survival in patients. In addition, it was found that LGALS1 was linked to oral cancer progression and metastasis and potentially regarded as a prognostic biomarker for oral cancer therapy [30].

Recent studies have shown that tumor-infiltrating immune cells can regulate tumor development and progression [31]. Previous studies have shown that AUNIP, a novel prognostic biomarker, has been shown to be related to stromal and immune scores in OSCC. It was demonstrated that AUNIP was associated with TME, human papillomavirus infection, and cell cycle in OSCC. The inhibited AUNIP led to the suppression of OSCC cell proliferation and brought about G0/G1 phase arrest in OSCC cells. The survival analysis showed that the overexpression of AUNIP predicted poor prognosis of OSCC patients [27–31]. Here, utilizing Cox regression analysis, we identified 3 immune cells with predictive significance in the prognosis of OSCC. The higher the risk score, the lower the level of immune cells in OSCC patients, and the worse the overall viability, suggesting that 3 immune cell function defects are closely related to the prognosis of oral squamous cell carcinoma patients. We observed that the infiltration level of myeloid dendritic cell, T cell CD4^+^ central memory, and common myeloid progenitor was strongly associated with better survival. Myeloid dendritic cells (MDCs), one of the major professional antigen-presenting cells, play a crucial determinant role in the immune responses to antigens [32]. The role of MDCs in OSCC has not been extensively studied. Recently, it is reported that memory T cells in tumors are an independent prognostic factor for several human malignancies [33, 34]. Memory T cells in the tumors may influence the development of lymphatic metastasis of OSCC [35]. Induction of memory T cells may be beneficial leading to cancer therapy. The common myeloid progenitor is a critical part in the immune regulation of tumors, where they are able to suppress both innate and adaptive immunity, including immunity to cancer [36]. While in the results of our analysis, high infiltration level of myeloid progenitor was associated with good prognosis in OSCC patients. Therefore, the clinical importance of these immune cells in OSCC may be ubiquitous.

Our study also has some limitations. First, our study mainly focused on the key genes and immune cells derived from Cox regression analysis, and other genes were not studied in depth. Second, we lacked clinical samples to validate the prognostic value of key genes and immune cells. More studies are needed to explore the novel therapeutic targets for OSCC for key genes and immune cells we identified. Finally, the Cox model we constructed will be validated in a large clinical cohort in the further.

## 5. Conclusion

Some of the genes found in this study already have prognostic significance and may be repositioned as targets for the treatment of OSCC. We used a bioinformatics approach to screen core genes and explore the impact of immune cell infiltration on the prognosis of OSCC patients. This study broadens the existing findings and reveals genes and immune cells associated with OSCC, opening the way for the development of novel targeted therapies.

## Figures and Tables

**Table 1:** Top 10 genes with smallest *P* values in Cox proportional hazards regression analysis.

Symbol	HR (exp(coef))	Coef	95% CI lower	95% CI upper	*Z*	*P* value
SERPINE1	1.433568	0.360166	0.170767	0.549565	3.72712	1.94*E* − 04
SCG5	1.297125	0.26015	0.11918	0.40112	3.616973	2.98*E* − 04
MUC15	0.770907	-0.26019	-0.40435	-0.11602	-3.53731	4.04*E* − 04
CD1C	0.528052	-0.63856	-0.99697	-0.28015	-3.49199	4.79*E* − 04
NEK6	1.976434	0.681294	0.292506	1.070083	3.434546	5.94*E* − 04
INHBA	1.43777	0.363093	0.152753	0.573433	3.383334	7.16*E* − 04
LAMC2	1.416473	0.34817	0.146206	0.550134	3.378821	7.28*E* − 04
CX3CR1	0.573106	-0.55668	-0.91193	-0.20144	-3.07136	0.002131
SPINK7	0.882734	-0.12473	-0.20696	-0.0425	-2.97293	0.00295
TGM3	0.885544	-0.12155	-0.20225	-0.04086	-2.95226	0.003155

## Data Availability

The data used during the present study are available from the corresponding author upon reasonable request.

## References

[1] Johnson D. E., Burtness B., Leemans C. R., Lui V. W. Y., Bauman J. E., Grandis J. R. (2020). Head and neck squamous cell carcinoma. *Nature Reviews. Disease Primers*.

[2] Fan H., Tian H., Cheng X. (2020). Aberrant Kank1 expression regulates YAP to promote apoptosis and inhibit proliferation in OSCC. *Journal of Cellular Physiology*.

[3] Chai A. W. Y., Yee P. S., Price S. (2020). Genome-wide CRISPR screens of oral squamous cell carcinoma reveal fitness genes in the Hippo pathway. *eLife*.

[4] Sano D., Myers J. N. (2007). Metastasis of squamous cell carcinoma of the oral tongue. *Cancer Metastasis Reviews*.

[5] Li Q., Dong H., Yang G., Song Y., Mou Y., Ni Y. (2020). Mouse tumor-bearing models as preclinical study platforms for oral squamous cell carcinoma. *Frontiers in Oncology*.

[6] Pai S., Bamodu O. A., Lin Y. K. (2019). CD47-SIRP*α* signaling induces epithelial-mesenchymal transition and cancer stemness and links to a poor prognosis in patients with oral squamous cell carcinoma. *Cell*.

[7] Palve V. C., Teni T. R. (2012). Association of anti-apoptotic Mcl-1L isoform expression with radioresistance of oral squamous carcinoma cells. *Radiation Oncology*.

[8] Peltanova B., Raudenska M., Masarik M. (2019). Effect of tumor microenvironment on pathogenesis of the head and neck squamous cell carcinoma: a systematic review. *Molecular Cancer*.

[9] Ribeiro Franco P. I., Rodrigues A. P., de Menezes L. B., Pacheco Miguel M. (2020). Tumor microenvironment components: allies of cancer progression. *Pathology, Research and Practice*.

[10] Peña-Oyarzún D., Reyes M., Hernández-Cáceres M. P. (2020). Role of autophagy in the microenvironment of oral squamous cell carcinoma. *Frontiers in Oncology*.

[11] Jiang Z., Wu C., Hu S. (2021). Research on neck dissection for oral squamous-cell carcinoma: a bibliometric analysis. *International Journal of Oral Science*.

[12] Hsu P. J., Yan K., Shi H., Izumchenko E., Agrawal N. (2020). Molecular biology of oral cavity squamous cell carcinoma. *Oral Oncology*.

[13] Sasahira T., Kirita T. (2018). Hallmarks of cancer-related newly prognostic factors of oral squamous cell carcinoma. *International Journal of Molecular Sciences*.

[14] Hanahan D., Weinberg R. A. (2011). Hallmarks of cancer: the next generation. *Cell*.

[15] Aran D., Hu Z., Butte A. J. (2017). xCell: digitally portraying the tissue cellular heterogeneity landscape. *Genome Biology*.

[16] Wang L. H., Xu M., Fu L. Q., Chen X. Y., Yang F. (2018). The antihelminthic niclosamide inhibits cancer stemness, extracellular matrix remodeling, and metastasis through dysregulation of the nuclear *β*-catenin/c-Myc axis in OSCC. *Scientific Reports*.

[17] Yin P., Su Y., Chen S. (2021). MMP-9 knockdown inhibits oral squamous cell carcinoma lymph node metastasis in the nude mouse tongue-xenografted model through the RhoC/Src pathway. *Analytical Cellular Pathology*.

[18] Zitvogel L., Galluzzi L., Kepp O., Smyth M. J., Kroemer G. (2015). Type I interferons in anticancer immunity. *Nature Reviews. Immunology*.

[19] Zhao C., Zhang G., Liu J., Zhang C., Yao Y., Liao W. (2020). Exosomal cargoes in OSCC: current findings and potential functions. *PeerJ*.

[20] Lu P., Takai K., Weaver V. M., Werb Z. (2011). Extracellular matrix degradation and remodeling in development and disease. *Cold Spring Harbor Perspectives in Biology*.

[21] Yang C. Y., Liu C. R., Chang I. Y. (2020). Cotargeting CHK1 and PI3K synergistically suppresses tumor growth of oral cavity squamous cell carcinoma in patient-derived xenografts. *Cancers*.

[22] Ge Y., Li W., Ni Q., He Y., Chu J., Wei P. (2019). Weighted gene co-expression network analysis identifies hub genes associated with occurrence and prognosis of oral squamous cell carcinoma. *Medical Science Monitor*.

[23] Fan Q. C., Tian H., Wang Y., Liu X. B. (2019). Integrin-*α*5 promoted the progression of oral squamous cell carcinoma and modulated PI3K/AKT signaling pathway. *Archives of Oral Biology*.

[24] Guo W., Giancotti F. G. (2004). Integrin signalling during tumour progression. *Nature Reviews. Molecular Cell Biology*.

[25] Dhanda J., Triantafyllou A., Liloglou T. (2014). SERPINE1 and SMA expression at the invasive front predict extracapsular spread and survival in oral squamous cell carcinoma. *British Journal of Cancer*.

[26] Salameti V., Bhosale P. G., Ames-Draycott A., Sipila K., Watt F. M. (2019). NOTCH1 signaling in oral squamous cell carcinoma via a TEL2/SERPINE1 axis. *Oncotarget*.

[27] Lin B., Li H., Zhang T., Ye X., Yang H., Shen Y. (2021). Comprehensive analysis of macrophage-related multigene signature in the tumor microenvironment of head and neck squamous cancer. *Aging (Albany NY)*.

[28] Hadler-Olsen E., Wirsing A. M. (2019). Tissue-infiltrating immune cells as prognostic markers in oral squamous cell carcinoma: a systematic review and meta-analysis. *British Journal of Cancer*.

[29] Luo W., Lin Y., Meng S., Guo Y., Zhang J., Zhang W. (2016). miRNA-296-3p modulates chemosensitivity of lung cancer cells by targeting CX3CR1. *American Journal of Translational Research*.

[30] Huang L., Ma B., Ma J., Wang F. (2017). Fractalkine/CX3CR1 axis modulated the development of pancreatic ductal adenocarcinoma via JAK/STAT signaling pathway. *Biochemical and Biophysical Research Communications*.

[31] Lv L., Zhao Y., Wei Q., Zhao Y., Yi Q. (2020). Downexpression of HSD17B6 correlates with clinical prognosis and tumor immune infiltrates in hepatocellular carcinoma. *Cancer Cell International*.

[32] Worbs T., Hammerschmidt S. I., Forster R. (2017). Dendritic cell migration in health and disease. *Nature Reviews. Immunology*.

[33] Galon J., Costes A., Sanchez-Cabo F. (2006). Type, density, and location of immune cells within human colorectal tumors predict clinical outcome. *Science*.

[34] Lee H. E., Chae S. W., Lee Y. J. (2008). Prognostic implications of type and density of tumour-infiltrating lymphocytes in gastric cancer. *British Journal of Cancer*.

[35] Enomoto K., Sho M., Wakatsuki K. (2012). Prognostic importance of tumour-infiltrating memory T cells in oesophageal squamous cell carcinoma. *Clinical and Experimental Immunology*.

[36] Kramer E. D., Abrams S. I. (2020). Granulocytic myeloid-derived suppressor cells as negative regulators of anticancer immunity. *Frontiers in Immunology*.

